# Cross-country abortion travel to England and Wales: results from a cross-sectional survey exploring people’s experiences crossing borders to obtain care

**DOI:** 10.1186/s12978-021-01158-z

**Published:** 2021-05-22

**Authors:** Camille Garnsey, Giulia Zanini, Silvia De Zordo, Joanna Mishtal, Alexandra Wollum, Caitlin Gerdts

**Affiliations:** 1 Ibis Reproductive Health, 1736 Franklin St, Suite 600, Oakland, CA 94612 USA; 2 Department of Anthropology, University of Barcelona, Montalegre, 6-8 08001 Barcelona, Spain; 3 Department of Anthropology, University of Central Florida, 4297 Andromeda Loop, Orlando, FL 32816 USA

**Keywords:** Europe, Induced abortion, Abortion travel, Abortion barriers

## Abstract

**Background:**

The laws governing abortion access vary across Europe. Even in countries with relatively liberal laws, numerous barriers to abortion access exist. In response to these barriers, evidence suggests that people living in countries with both restrictive and liberal laws travel outside of their home country for abortion care. England and Wales are common destinations for those who travel to seek abortions, but little is known about the motivations and experiences of those who undertake cross-country travel to England or Wales to obtain care. This paper aims to describe the abortion seeking and travel experiences of women and pregnant people who traveled to England and Wales for an abortion between 2017 and 2019.

**Methods:**

We recruited 97 participants who had traveled cross-country from both liberal and restrictive contexts to seek abortion care at three participating BPAS clinics in England and Wales. Participants completed an electronic survey about their reproductive histories, abortion decision-making, experiences seeking abortion care, and traveling. We conducted a descriptive analysis, and include comparisons between participants who traveled from liberal and restrictive contexts.

**Results:**

Over a third of participants considered abortion four weeks or more before presenting for care at BPAS, and around two-thirds sought abortion services in their home country before traveling. The majority of participants indicated that they would have preferred to have obtained an abortion earlier and cited reasons including scheduling issues, a dearth of local services, delayed pregnancy recognition, and financial difficulties as causing their delay. About seventy percent of participants reported travel costs between €101–1000 and 75% of participants reported that the cost of the abortion procedure exceeded €500. About half of participants indicated that, overall, their travel was very or somewhat difficult.

**Conclusions:**

This analysis documents the burdens associated with cross-country travel for abortion and provides insight into the factors that compel people to travel. Our findings highlight the need for expanded access to abortion care throughout Europe via the removal of legal impediments and other social or procedural barriers. Removing barriers would eliminate the need for cumbersome abortion travel, and ensure that all people can obtain necessary, high-quality healthcare in their own communities.

## Plain language summary

In Europe, people who live in countries where abortion is severely restricted or illegal altogether lack access to abortion care entirely, but even people who live in countries with more liberal laws face barriers due to gestational age limits, waiting periods, and a lack of trained and willing providers. Existing evidence suggests that restrictions and barriers compel people from both countries with restrictive laws as well as those from countries with more liberal laws to travel outside of their home country for abortion services. England and Wales are common destinations for people traveling within Europe to obtain abortion services, but little is known about the experiences of these travelers. We surveyed individuals who had traveled from another country to seek abortion services in England or Wales. Our analysis documents that many participants contemplated getting an abortion and sought care in their home countries before traveling. Likewise, many participants indicated that they would have preferred to have obtained an abortion earlier in their pregnancy, and referenced scheduling issues, a dearth of local services, delayed pregnancy recognition, and financial difficulties as causing their delay. A majority of participants indicated that covering the costs of their abortion, and the costs of travel was difficult, and that the travel experience in its entirety was difficult. Our findings document the reasons for, and burdens associated with abortion travel and highlight the need to expand access to abortion across Europe via the elimination of all legal restrictions and impediments.

## Introduction

Access to legal abortion remains fragmented across Europe. In countries with laws that permit abortion on broad social or economic grounds, including Germany, France, Italy, and Belgium, abortion access is constrained by gestational age limits, waiting periods, and a lack of trained and willing providers [1–3]. In Poland, Malta, and, until the 2019 implementation of the new abortion law, the Republic of Ireland the grounds for legal abortion have been severely limited, or abortion has been completely illegal [4]. Existing evidence suggests that women and pregnant people living in countries with highly restrictive abortion laws, as well as those living in countries with relatively liberal laws that nevertheless over-regulate the provision of abortion services may be compelled by any number of the aforementioned barriers to travel across borders to seek legal care [5, 6].

The laws governing abortion in Great Britain, while among the most liberal in Europe, continue to overregulate the provision of abortion. Nevertheless, with abortion permitted on broad social or economic grounds until 24 weeks of pregnancy [4] and the proximity of Great Britain to countries in Europe with more restrictive policies, parts of Great Britain, specifically England and Wales, are common destinations for people seeking abortion who travel due to limited access in their home countries. In 2018, 3,132 non-British European residents traveled to England and Wales to obtain abortion care. An estimated 95% of the foreign women who received care in 2018 had traveled from the Republic of Ireland prior to the 2018 change in the abortion law and the rest traveled from Malta, Poland, Italy, France, Germany and Denmark [7].

Little is known about the experiences of women and pregnant people^1^ who travel for abortion within Europe. A systematic review by Barr-Walker and colleagues[6] found that of 59 studies relevant to abortion travel, only 8 examined travel in Europe [5, 8–14], and even fewer studies have focused specifically on Great Britain, surveyed abortion travelers about their experiences, or documented the burdens they faced. One exploratory study conducted in 2015 assessed the feasibility of recruiting women who had traveled abroad to seek abortions at three British Pregnancy Advisory Service (BPAS) clinics in England and Wales [5]. In their preliminary quantitative exploration of the abortion seeking and travel experiences of study participants, the authors found that participants had traveled to England or Wales from 14 countries in Europe for reasons including the illegality of abortion in their home country and gestational age restrictions, and many found the travel difficult due to the associated costs [5]. The exploratory study offered preliminary insight into the phenomenon of abortion travel to England and Wales from other European countries. However, given the relative paucity of data on the experiences of those traveling for abortion in Europe, more research was needed.

Following the completion of the exploratory study, we launched a European Research Council (ERC) funded multi-country, 5 year, mixed-methods study led by Dr. Silvia De Zordo (Universitat de Barcelona) on the causes, consequences and experiences associated with abortion travel in Europe (BAR2LEGAB, 680,004—https://europeabortionaccessproject.org/). The study involved quantitative and qualitative data collection on cross-country abortion travel in the Netherlands, Spain, and England and Wales, and in-country abortion travel in France, Italy, and Spain. For the cross-country portion of the study, we sought to document the experiences of people who travel for abortion care from across Europe to countries with later gestational age limits, and to compare the experiences of those who traveled from more liberal contexts to the experiences of those who traveled from highly restrictive environments. The present analysis draws on data collected as part of this larger study and aims to describe the abortion seeking and travel experiences of women and pregnant people who traveled to England or Wales for abortion care between 2017 and 2019.

## Methods

In this paper, we present findings from quantitative surveys fielded at three BPAS clinics in England and Wales between July 2017 and March 2019. Clinics were selected as data collection sites based on available information about the annual volume of non-residents who sought abortion care at each clinic in the years prior to the launch of the study.

People seeking abortions were eligible for inclusion in our study if they were 18 years of age or older, had traveled from any non-UK country in the European Union to seek abortion care at one of the participating clinics, and were proficient in French, Italian, English, German, or Spanish, as these were the languages in which the survey was available. Eligible individuals were identified by researchers on the study team and/or non-care providing BPAS clinic staff. Researchers approached eligible individuals with information about the study in the clinic waiting rooms. Those who expressed interest in participating were offered the option to complete an anonymous, self-administered, tablet-based survey and/or to take part in a confidential in-depth interview with a researcher before the initiation of their abortion procedure. Only results from the survey portion of the study are presented in this paper.

Researchers recruited participants traveling from countries with relatively liberal abortion laws and participants traveling from countries with restrictive laws. The multi-country ERC-funded study from which this analysis is derived was focused on  describing the experiences of women and pregnant people traveling for abortion from relatively liberal contexts within Europe. Our recruitment efforts, therefore, were designed to over-sample travelers from countries with relatively liberal laws, as well as to recruit a sample of travelers from restrictive contexts large enough to conduct a comparative analysis. The goal for the multi-country study was to have a final sample with a 2:1. ratio of travelers from relatively liberal contexts to travelers from restrictive contexts. For the purpose of recruitment and analysis, we define countries as having relatively liberal abortion laws if abortion was available on request, or on broad grounds within legally specified gestational age (GA) limits throughout our data collection period. Countries classified in our analysis as having relatively liberal laws were Germany, France, Italy, Belgium, Austria, Luxemburg, Bulgaria, and Denmark. Malta and Poland were considered to be countries with restrictive laws, as abortion was only permitted in narrowly specified cases including in the cases of rape, incest, severe fetal anomalies, or risk to the health or life of the pregnant person, or banned altogether throughout our data collection period. The Republic of Ireland was also categorized as a country with a restrictive law until January 1, 2019 when abortion access was liberalized to include services available on request for up to 12 weeks of gestation. No travelers from the Republic of Ireland (or any other country with restrictive laws) were recruited for our study after December of 2018 because the lower than anticipated volume of travelers from relatively liberal contexts during our study period meant that travelers from restrictive contexts were already overrepresented in our sample. Therefore, no Irish participants were recruited following the change in the abortion law and all travelers from the Republic of Ireland are classified as having traveled from a restrictive context in this analysis.

Surveys were primarily administered via tablets at the clinic. In an effort to boost recruitment, part-way through the data collection period we added the option for participants who either did not have time to complete the survey at the clinic or preferred to complete the survey at a later date to provide their contact information and have the survey sent to them via text or email for completion at a later time. We continued to administer and collect surveys in the clinic after instituting this addition to the protocol, and ultimately, only two travelers opted to complete the survey remotely. Consent was collected electronically prior to survey initiation for all participants. Participants who completed the survey in the clinic were remunerated with €10 and participants who completed the survey remotely were sent a €10 gift card. 106 out of 199 total travelers informed about the study completed the survey, yielding a robust response rate of 53%. Nine completed surveys were ultimately deemed ineligible and are excluded from this analysis.

We collected data on the sociodemographic profile of travelers, pregnancy detection and decision-making, participants’ experiences seeking care in their home-country, and their reasons for and experiences with traveling. For reasons for traveling, participants were asked to disclose any and all reasons that they decided to travel for abortion. If participants selected just one reason, that reason was categorized as the participant’s main reason for traveling. If participants selected more than one reason, they were asked to pick a main reason from the list of reasons they had selected. For this analysis, we present only the main reasons participants traveled. Participants were asked to report their travel and accommodation costs separately, but costs are summed in our analysis.^2^

For this paper, we analyze variables descriptively and present responses broken out by participants’ legal environment. However, due to the relatively small sample of those who traveled from countries with more liberal laws, we do not present statistical tests to assess differences between the two groups. We do highlight notable descriptive differences between the groups where applicable but acknowledge that research with larger samples would be necessary in order to draw statistically robust conclusions. Unless noted in the tables, all percentages reported are out of the total number of participants in each group, and the proportion of missing responses is calculated and presented in the tables for each variable. Analyses were conducted using STATA 15 SI. This arm of the larger study received ethical approval from the ERC Ethics Committee and the BPAS Research & Ethics Committee.

## Results

Ninety-seven travelers participated in the survey. Twenty-six percent (n = 25) of recruited participants traveled from countries with relatively liberal laws including Italy (n = 11), France (n = 5), Denmark (n = 4), Germany (n = 2), Belgium (n = 1), Bulgaria (n = 1), and Austria (n = 1). The remaining three-quarters (n = 72) of participants traveled from countries with highly restrictive laws, with the vast majority coming from The Republic of Ireland (n = 65), and a few participants each traveling from Malta (n = 4), and Poland (n = 3).

### Sociodemographic profile of participants

Table 1 provides the demographic profile of participants. Over a third of participants in our sample were between the ages of 18 and 24 (37%), 42% were aged 25–34, and the remaining 21% were 35 or older. The majority of participants had completed university (61%), were employed in some capacity (63%), and had sufficient resources to meet their basic needs all or most of the time (83%). About half of all travelers were either single, separated, or divorced (53%), and 43% reported being married or in a civil partnership. Most participants identified as either Catholic (61%), or Atheist/Agnostic/not identifying with a religion (28%). We did observe a few notable descriptive differences between the sociodemographic characteristics of participants who had traveled from liberal contexts compared to those who had traveled from restrictive contexts, specifically that a lower proportion of participants from liberal contexts reported being employed full time (44% vs. 70%) and having sufficient income to meet their basic needs all of the time (78% vs. 96%).

**Table 1:** Sociodemographic characteristics

	Women who traveled from restrictive contexts (n = 72) N (%)	Women who traveled from liberal contexts (n = 25) N (%)	All travelers (n = 97) N (%)
Country of residence			
France	NA	5 (20%)	5 (5%)
Italy	NA	11 (44%)	11 (11%)
Germany	NA	2 (8%)	2 (2%)
Belgium	NA	1 (4%)	1 (1%)
Austria	NA	1 (4%)	1 (1%)
Denmark	NA	4 (16%)	1 (1%)
Bulgaria	NA	1 (4%)	1 (1%)
The Republic of Ireland	65 (98%)	NA	65 (67%)
Malta	4 (6%)	NA	4 (4%)
Poland	3 (4%)	NA	3 (3%)
Highest level of education completed			
Secondary school or below	18 (25%)	5 (20%)	23 (24%)
Some university	8 (11%)	6 (24%)	14 (14%)
University or graduate school	45 (63%)	13 (52%)	58 (60%)
Prefer not to answer/no response	1 (1%)	1 (4%)	2 (2%)
Age			
18–24	26 (36%)	10 (40%)	36 (37%)
25–34	31 (43%)	10 (40%)	41 (42%)
35–46	15 (21%)	5 (20%)	20 (21%)
Prefer not to answer/no response	0	0	0
Employment			
Employed full-time, part-time, or self-employed	49 (68%)	11 (44%)	60 (62%)
Unemployed	8 (11%)	0	8 (8%)
Student	10 (14%)	9 (36%)	19 (20%)
Other	3 (4%)	5 (20%)	8 (8%)
Prefer not to answer/no response	2 (3%)	0	2 (2%)
Ability to meet basic needs			
All or most of the time	54 (75%)	23 (92%)	77 (79%)
Some of the time	10 (14%)	0	10 (10%)
Never or rarely	5 (7%)	1 (4%)	6 (6%)
Prefer not to answer/no response	3 (4%)	1 (4%)	4 (4%)
Marital status			
Married or in a civil partnership	28 (39%)	13 (52%)	41 (42%)
Single, separated, or divorced	39 (54%)	12 (48%)	51 (53%)
Other	4 (6%)	0	4 (4%)
Prefer not to answer/no response	1 (1%)	0	1 (1%)
Religious Affiliation			
Catholic	44 (61%)	13 (52%)	57 (59%)
Atheist, Agnostic, or doesn’t identify with a religion	21 (29%)	5 (20%)	26 (27%)
Other	5 (7%)	6 (24%)	11 (11%)
Prefer not to answer/no response	2 (3%)	1 (4%)	3 (3%)

### Reproductive history and experiences seeking abortion care in country of residence

Information about participants’ reproductive histories, and experiences seeking abortion care in their countries of residence are provided in Table 2. Just under two-thirds of participants had no children (62%), and most had never had a prior abortion (85%). Participants in our study reported presenting for abortion care in England or Wales at an average of 12.5 weeks of gestation (e.g., 12 weeks + 4 days). While sample sizes are small, there was a notable difference in mean weeks of gestation when presenting for services between people who had traveled from restrictive contexts, who presented at an average of 10.7 weeks (e.g., 10 weeks and 5 days), and those who traveled from more liberal contexts, who presented at an average of 18.1 weeks of gestation (e.g.,18 weeks and 1 day).

**Table 2:** Reproductive history and experiences seeking abortion care

	Women who traveled from restrictive contexts (n = 72) N (%)	Women who traveled from liberal contexts (n = 25) N (%)	All travelers (n = 97) N (%)
Number of children			
0	46 (64%)	14 (56%)	60 (62%)
1–2	21 (29%)	10 (40%)	31 (32%)
3 +	5 (7%)	1 (4%)	6 (6%)
Prefer not to answer/no response	0	0	0
Prior abortion			
Yes	9 (13%)	5 (20%)	14 (14%)
No	62 (86%)	20 (80%)	82 (85%)
Prefer not to answer/no response	1 (1%)	0	1 (1%)
Weeks of gestation when presenting for services			
1–12 weeks	48 (67%)	2 (8%)	50 (52%)
12–14 weeks	5 (7%)	0	5 (5%)
14–20 weeks	9 (13%)	11 (44%)	20 (21%)
20 weeks or more	7 (10%)	11 (44%)	18 (19%)
Prefer not to answer/no response	3 (4%)	1 (4%)	4 (4%)
Mean weeks of gestation when presenting for services	10.7 weeks	18.1 weeks	12.6 weeks
When first considered abortion			
1 week before presenting for care in England or Wales	9 (13%)	1 (4%)	10 (10%)
2–3 weeks before presenting for care in England or Wales	29 (40%)	14 (56%)	43 (44%)
4–5 weeks before presenting for care in England or Wales	19 (26%)	2 (8%)	21 (22%)
6 weeks or more before presenting for care in England or Wales	10 (14%)	6 (24%)	16 (16%)
Prefer not to answer/no response	5 (7%)	2 (8%)	7 (7%)
Sought abortion in country of residence before traveling			
Yes	39 (54%)	23 (92%)	62 (64%)
No	33 (46%)	2 (8%)	35 (36%)
Prefer not to answer/no response	0	0	0
Weeks first sought abortion in country of residence (if applicable, n = 62)**			
1 week before presenting for care in England or Wales	5 (13%)	2 (9%)	7 (11%)
2–3 weeks before presenting for care in England or Wales	20 (51%)	11 (48%)	31 (50%)
4–5 weeks before presenting for care in England or Wales	7 (18%)	2 (9%)	9 (15%)
6 + weeks before presenting for care in England or Wales	5 (13%)	7 (30%)	12 (19%)
Prefer not to answer/no response	2 (5%)	1 (4%)	3 (5%)
Attempted to self-manage an abortion prior to traveling			
Yes	7 (9%)	0	7 (7%)
No	62 (86%)	25 (100%)	87 (90%)
Prefer not to answer/no response	3 (4%)	0	3 (3%)
Preferred to obtain abortion earlier			
Yes	57 (80%)	22 (88%)	79 (82%)
No	9 (13%)	2 (8%)	11 (12%)
Reasons for delays in care-seeking^a^			
Issues with scheduling (both personal and getting an appointment at the clinic)	23 (32%)	8 (32%)	31 (32%)
No local abortion services	26 (36%)	4 (16%)	30 (31%)
Delayed pregnancy recognition	17 (24%)	12 (48%)	29 (30%)
Issues arranging travel	20 (28%)	5 (20%)	25 (26%)
Issues with money to pay for abortion or travel	17 (24%)	2 (8%)	19 (20%)
Abortion decision making	14 (19%)	5 (20%)	19 (20%)
Didn’t know where to get an abortion	8 (11%)	4 (16%)	12 (12%)
Change of situation	5 (7%)	5 (20%)	10 (10%)
Other	9 (13%)	2 (8%)	11 (11%)
No delays	9 (13%)	2 (8%)	11 (12%)
Prefer not to answer/no response			3 (3%)

^a^More than one answer was possible, percentages may exceed 100%

Thirty-eight percent of participants first considered abortion four weeks or more before presenting for care at BPAS. Seven participants (7% of the sample), all from restrictive contexts, reported trying to end their pregnancies on their own without medical supervision. Sixty-four percent of participants sought abortion services in their home country before traveling to England or Wales, and the majority of those who sought services did so 1–3 weeks before presenting for care in England or Wales. Compared to those who traveled from restrictive contexts, a greater proportion of participants who traveled from countries with liberal laws reported seeking care in their country of residence prior to travel (92% vs. 54%).

Eighty-two percent of all participants indicated that they would have preferred to have obtained an abortion earlier in their pregnancy and cited issues such as scheduling, a lack of local services, delayed pregnancy recognition, difficulties associated with paying for the abortion or travel, and decision-making factors as reasons for the delay. Many participants cited more than one factor as delaying their process. Compared to those from restrictive contexts, a higher proportion of participants from liberal contexts reported not getting their abortion when they wanted due to delayed pregnancy recognition (24% vs. 48%).

### Decision-making and information-gathering

Table 3 presents data on decision-making, including disclosure of the decision to have an abortion, and information-gathering. Forty-four percent of participants indicated it was somewhat or very difficult to reach their decision to have an abortion, 19% indicated that it was neither easy or difficult, and 32% indicated it was somewhat or very easy. Over half of participants (56%) indicated that they had kept their abortion secret from someone they wished they could have told, but only 9% reported that they had told someone that they did not want to. In detailing the various sources from which they obtained information about abortion services in Great Britain^3^, two thirds of participants said they got information from the internet (66%), about a quarter indicated that they got information from friends, family members, or partners (24%), and about a fifth referenced healthcare providers (19%). Participants learned about the specific BPAS clinics through similar channels.

**Table 3:** Experiences with abortion decision-making and information seeking

	Women who traveled from restrictive contexts (n = 72) N (%)	Women who traveled from liberal contexts (n = 25) N (%)	All travelers (n = 97) N (%)
Difficulty of reaching decision to have abortion			
Somewhat or very easy	23 (32%)	8 (32%)	31 (32%)
Neither easy nor difficult	12 (17%)	6 (24%)	18 (19%)
Somewhat or very difficult	34 (47%)	9 (36%)	43 (44%)
Prefer not to answer/no response	3 (4%)	2 (8%)	1 (1%)
Kept abortion secret from someone participants wished to tell			
Yes	40 (56%)	14 (56%)	54 (56%)
No	30 (42%)	10 (40%)	40 (41%)
Prefer not to answer/no response	2 (3%)	1 (4%)	3 (3%)
Told someone participants didn’t want to tell			
Yes	6 (8%)	3 (12%)	9 (9%)
No	65 (90%)	21 (84%)	86 (89%)
Prefer not to answer/no response	1 (1%)	1 (4%)	2 (2%)
Primary reason for traveling			
Abortion is not legal in country of residence	68 (94%)	1 (4%)	69 (71%)
Too late to have abortion in country of residence	0	18 (72%)	18 (19%)
It was difficult to find a physician willing to provide care or I was worried about a health provider refusing to help me	1 (1%)	2 (8%)	3 (3%)
I was worried about someone finding out about my abortion	0	1 (1%)	1 (1%)
I didn’t know where to get an abortion in my country of residence or there are no abortion services where I live	3 (4%)	2 (8%)	5 (5%)
I wanted to have a surgical termination, which is not available in my country	0	1 (4%)	1 (1%)
Prefer not to answer/no response	0	0	0
Sources of information about abortion services in the Britain ^a^			
Websites	52 (72%)	12 (48%)	64 (66%)
Friend, family, or partners	21 (29%)	2 (8%)	23 (24%)
Health care providers	12 (17%)	6 (24%)	18 (19%)
Media	6 (8%)	3 (12%)	9 (9%)
Other	7 (10%)	2 (8%)	9 (9%)
Prefer not to answer/no response	–	–	8 (8%)
Sources of information about BPAS clinics^a^			
Media	3 (4%)	2 (8%)	5 (5%)
Websites	46 (64%)	8 (32%)	54 (56%)
Friends, family, or partners	12 (17%)	3 (12%)	15 (15%)
Health care providers	16 (22%)	7 (28%)	23 (24%)
Other	8 (11%)	1 (4%)	9 (9%)
Prefer not to answer/no response			9 (9%)
Reason for traveling to Britain specifically^a^			
It was the easiest to get to	45 (63%)	8 (32%)	53 (55%)
It was the closest country that provided abortion at my gestation	26 (36%)	10 (40%)	36 (37%)
Recommendations from providers, friends, or someone else	17 (24%)	4 (16%)	21 (22%)
It was the cheapest country to get to	9 (13%)	1 (4%)	10 (10%)
I know someone in Britain	4 (6%)	5 (20%)	9 (9%)
Clinics in Britain offered the least expensive abortions	2 (3%)	1 (4%)	3 (3%)
Clinics in Britain offered surgical abortion	3 (4%)	6 (24%)	9 (9%)
Prefer not to answer/no response			2 (2%)

^a^More than one answer was possible, percentages may exceed 100%

Participants’ primary reason for traveling to England and Wales differed by the restrictiveness of the contexts from which they traveled. Participants who traveled from restrictive contexts overwhelmingly indicated that they traveled primarily because abortion was not legal in their country of residence (94%), and a sizeable majority of participants from liberal contexts said they traveled because it was too late for them to have an abortion in their country of residence (72%). When asked why they traveled to the UK instead of somewhere else, participants gave reasons including that it was the easiest place to get to (55%), it was the closest place that provided abortion at their gestational age (37%), and providers, friends, or family members had recommended it (22%). Greater proportions of travelers from restrictive contexts reported that they traveled to the UK because it was the easiest or cheapest country to get to compared to travelers from liberal contexts, who cited the availability of surgical abortion or the fact that they knew someone in the UK more frequently.

### Travel costs and experiences

Participants’ travel experiences are detailed in Table 4. Ninety percent of participants’ journeys to England or Wales included air travel, and 15% of participants relied on more than one method of transportation. The majority of participants made the trip with a companion (74%), and most, including all of the participants who traveled from more liberal contexts, stayed in England or Wales for at least one night (79%). About half (53%) of participants indicated that, overall, their travel was very or somewhat difficult. Forty-nine percent of participants reported combined travel and accommodation costs between €101–500, an additional 21% reported costs between €501–1000 and another 10% reported costs exceeding €1000. Almost half of participants (44%) from restrictive contexts, and 84% of those that traveled from liberal contexts reported that the cost of the abortion procedure exceeded €1000. Participants covered the costs of their travel and abortion in a number of ways, including using savings (37%), receiving assistance from a friend or relative (27%), receiving assistance from a partner (24%), or putting off other expenses (16%). Twenty-nine percent of participants needed over a week to raise the money to cover the costs of their travel and/or abortion procedure, 31% needed under a week, and 30% did not need to raise money to cover the costs. Sixty-six percent of participants (66%) had to take time off of work to go to their appointment, and thirty-one percent of those who had to take time off lost wages. Sixty-nine percent of participants said it was somewhat or very difficult to cover the cost of travel, and a similar proportion (76%) indicated it was very or somewhat difficult to cover the cost of their abortion.

**Table 4:** Means of travel, travel costs, and travel experiences

	Women who traveled from restrictive contexts (n = 72) N (%)	Women who traveled from liberal contexts (n = 25) N (%)	All travelers (n = 97) N (%)
Mode of transportation for travel to Britain^a^			
Airplane	65 (90%)	22 (88%)	87 (90%)
Train	11 (15%)	5 (20%)	16 (16%)
Bus	5 (7%)	1 (4%)	6 (6%)
Personal Car	4 (6%)	0	4 (4%)
Other	3 (4%)	0	3 (3%)
Prefer not to answer/no response			1 (1%)
Used more than one mode of transportation to travel to Britain			
Yes	12 (17%)	3 (12%)	15 (15%)
No	60 (83%)	22 (88%)	82 (85%)
Prefer not to answer/no response	0	0	0
Travel accompaniment			
Traveled alone	17 (24%)	6 (24%)	23 (24%)
Traveled with a companion	53 (74%)	19 (76%)	72 (74%)
Prefer not to answer/no response	2 (3%)	0	2 (2%)
Stayed overnight			
Yes	52 (72%)	25 (100%)	77 (79%)
No	18 (26%)	0	18 (19%)
Prefer not to answer/no response	2 (3%)	0	2 (2%)
Total travel and accommodation costs			
€0	7 (10%)	4 (16%)	11 (11%)
€1–100	7 (10%)	0	7 (7%)
€101–500	39 (54%)	9 (36%)	48 (49%)
€501–1000	14 (19%)	6 (24%)	20 (21%)
More than €1001	5 (7%)	6 (24%)	11 (11%)
Prefer not to answer/no response	0	0	0
Cost of abortion procedure			
€350–500	22 (31%)	0	22 (23%)
€501–1000	25 (35%)	4 (16%)	29 (30%)
More than €1001	23 (32%)	21 (84%)	44 (45%)^a^
I did not have to pay for my abortion	2 (3%)0	0	2 (2%)
Prefer not to answer/no response	0	0	0
Time needed to cover the cost of traveling and abortion procedure			
Less than a week	21 (29%)	9 (36%)	30 (31%)
1–4 weeks	19 (26%)	3 (12%)	22 (23%)
Over 4 weeks	5 (7%)	1 (4%)	6 (6%)
I didn’t have to raise money	20 (28%)	9 (36%)	29 (30%)
Prefer not to answer/no response	7 (10%)	3 (12%)	10 (10%)
Ways in which participants covered the costs of traveling and/or the abortion^a^			
Delayed/put off other expenses	14 (19%)	2 (8%)	16 (16%)
Assistance from a friend or relative	17 (24%)	10 (40%)	27 (28%)
Assistance from partner	16 (22%)	7 (28%)	23 (24%)
Assistance from abortion fund	5 (7%)	0	5 (5%)
Used savings	26 (36%)	10 (40%)	36 (37%)
Used a credit card or received credit from a bank	10 (14%)	0	10 (10%)
Other	5 (7%)	1 (4%)	6 (6%)
Prefer not to answer	3 (4%)	1 (4%)	4 (4%)
Difficulty of covering travel costs			
Very or somewhat easy	18 (25%)	8 (32%)	26 (27%)
Very or somewhat difficult	51 (71%)	16 (64%)	67 (69%)
Prefer not to answer/no response	3 (4%)	1 (4%)	4 (4%)
Difficulty of covering abortion cost			
Very or somewhat easy	12 (17%)	6 (24%)	18 (19%)
Very or somewhat difficult	56 (78%)	18 (72%)	74 (76%)
I did not have to pay for my abortion	2 (3%)	0	2 (2%)
Prefer not to answer/no response	2 (3%)	1 (4%)	3 (3%)
Overall difficulty of traveling			
Very or somewhat easy	31 (43%)	14 (56%)	45 (46%)
Very or somewhat difficult	40 (56%)	11 (44%)	51 (53%)
Prefer not to answer/no response	1 (1%)	0	1 (1%)
Time off of work			
Yes	49 (68%)	15 (60%)	64 (66%)
No	19 (26%)	10 (40%)	29 (30%)
Prefer not to answer/no response	4 (6%)	0	4 (4%)
Lost Wages (if time off work, n = 64)**			
Yes	16 (33%)	4 (27%)	20 (31%)
No	30 (61%)	9 (60%)	39 (61%)
Prefer not to answer/no response	3 (6%)	2 (13%)	5 (8%)

^a^More than one answer was possible, percentages may exceed 100%

**Total n the number of participants who indicated that they had to take time off of work

## Discussion

Findings from our study contribute to an emerging body of literature demonstrating that women and pregnant people who live in countries across Europe that restrict the timeframe and circumstances under which legal abortion can be obtained undergo burdensome travel to seek care in England or Wales. This analysis expands our understanding of the phenomena of abortion travel to Britain from countries across Europe, and offers a more in-depth look at individuals’ decision-making processes and the burden of travel.

This paper provides new insight into travelers’ reproductive experiences and abortion care-seeking in their country of residence. We found that many participants considered abortion well before they presented for care at a BPAS clinic, that the majority sought abortion care in their country of residence before traveling, some attempted to end their pregnancy on their own, and most would have preferred to obtain an abortion at an earlier stage in their pregnancy. While our sample is too small to allow us to assess whether differences in the reproductive histories and in-country care seeking experiences of those that traveled from more restrictive contexts differed significantly from those who came from liberal contexts, we did document notable descriptive differences between these two categories of travelers. Compared to those who traveled from restrictive contexts, travelers who came from liberal contexts presented for care at BPAS at a later gestation. Participants from liberal contexts also reported delayed pregnancy recognition more frequently than those who traveled from restrictive contexts, a factor that has been consistently associated in the literature with later presentation for abortion in other places [15–17]. When considered alongside recently published findings suggesting that most people who travel cross-country for abortion from liberal contexts do so because of gestational age limits [18], these findings highlight the negative impact of gestational limits on abortion access. They also demonstrate that, regardless of whether other barriers are removed, a subset of people will always exceed gestational limits due to delayed pregnancy recognition. It is thus imperative for countries across Europe to remove gestational age limit laws.

Our data brought to light another key difference between travelers from restrictive contexts and liberal legal contexts: a greater proportion of participants who traveled from liberal contexts sought care in their home country before traveling, and thus spent at least some time seeking care at home. This difference may exist because women and pregnant people from liberal contexts believe, due to the broader legal status of abortion in their country, that they would/should be able to access an abortion at home, and thus spend time and energy seeking and arranging in-country care only to find that they cannot obtain services due to gestational age limits[18]. On the other hand, women and pregnant people from restrictive contexts might be more immediately aware that they cannot obtain legal services at home and consequently spend less time searching for care and make their travel arrangements more quickly after discovering they are pregnant. It is also possible that widespread knowledge about the lack of available abortion services in restrictive contexts may explain our finding that all participants in our sample who reported attempting to end their pregnancy on their own before traveling resided in countries where abortion is severely legally restricted. Further research is needed to explore how knowledge of local abortion laws influences decision making and impacts delays in care seeking.

Despite what we found with regards to gestation at the time of presentation and in-country care seeking, similar proportions of participants from both groups indicated that they would have preferred to obtain their abortion earlier. The reasons participants from both contexts gave for their delays paint a picture of care-seeking processes stymied by obstacles to care and/or complicated by the myriad and sometimes dynamic financial, interpersonal, emotional and logistical factors that influence the decisions people make about their reproductive health. Participants in our study, regardless of country of residence, cited multiple reasons why they were not able to get their abortions when they wanted, including issues with finding care, arranging or paying for travel, and slowdowns related to decision making. Other studies exploring abortion travel have documented the impact of similar factors on people’s ability to obtain timely care[17, 19–21].

Our study also provides a greater understanding of the cost, logistics, and burdens associated with cross-country travel for abortion. Most participants in our sample had to take time off of work, fly to England or Wales, stay overnight, spend €100–1000 on accommodations and upwards of €500 on their abortion procedure (an expense not documented in the 2016 study). A majority of participants did not have the funds on-hand to cover these expenses, and needed time to raise the money to pay for their travel and abortion. It is also worth noting that while the substantial majority of participants reported that it was difficult to cover the costs of travel (69%) and the costs of their abortion procedure (76%), respectively, a far smaller proportion of participants (53%) reported that they found the overall experience of traveling for abortion—of which cost was one component—to be difficult. Previous abortion research has documented that abortion seekers at times describe certain aspects of their abortion experience negatively while rating the overall experience favorably [22–26]. These studies have suggested that self-reports of overall satisfaction with an abortion experience may be strongly linked to achievement of the desired outcome of no longer being pregnant, or that shame and stigma may play a role in giving patients such low expectations that even substandard care exceeds expectations [22–26]. It is possible that our findings represent an analogous phenomenon with regards to participants’ reports of overall difficulty being influenced by the ultimate achievement of their desired outcome of pregnancy termination. It is also possible that certain aspects of the abortion process were more or less difficult than others, and that participants focused on the more positive elements of their experience when answering the overall questions. In either case, it is clear that travel for abortion care represents a significant burden and participants had to interrupt their daily activities, and raise and invest considerable resources into ending their pregnancy. While everyone in our study ultimately received the care they wanted, the logistical and financial burdens associated with travel that were encountered by participants in our study, have in previous studies, been hypothesized to contribute to an increase in unwanted pregnancies being carried to term [13, 17, 20], an outcome that can incur a host of adverse consequences for parents and families [27].

Half of participants in our sample kept their abortion a secret from someone they wished they had told, and 10% told someone they did not want to. This finding allows us to integrate interpersonal barriers, and their associated logistical challenges, into our understanding of the burdens associated with abortion travel. Participants may have kept their abortions secret for fear of being judged, stigmatized, or having their decision challenged or obstructed; previous studies have documented that such fears can influence who people tell about their pregnancies, which has consequences for who they are able to rely on for both practical and emotional support [28–30].

It is important to highlight a number of limitations to our analysis. First, our sample cannot be considered representative of all people traveling for abortion care in England or Wales, nor all of those traveling to BPAS clinics. In addition, despite our efforts to over-sample travelers from countries with relatively liberal abortion laws, the low volume of people traveling during our recruitment period meant that we were unable to successfully achieve a 2:1 ratio of travelers from liberal and restrictive setting. This resulted in the overrepresentation of travelers from the Republic of Ireland, further limiting the representativeness of our sample, even among travelers from restrictive settings specifically, and hindering our ability to conduct statistically comparative analyses. However, despite the fewer than anticipated travelers from countries other than the Republic of Ireland, the mix of travelers from restrictive and legal contexts that were recruited for our study enable us to make a unique contribution to the literature on abortion travel. Additionally, our data on the cost of travel may be an underestimate of the actual costs participants incurred, as our questions did not clearly ask participants to report round-trip costs. In addition, while our analysis documents the obstacles and burdens faced by individuals who traveled to BPAS clinics in England or Wales for abortion care, we are unable to describe the experiences of those women and pregnant people who ultimately found the obstacles and burdens associated with travel insurmountable and instead carried an unwanted pregnancy to term, successfully self-managed their abortion, or otherwise sought abortion outside of the formal healthcare system. Finally, although we found descriptive differences between the experiences of those who traveled from countries with restrictive vs. liberal abortion laws, we are not able to assess whether the differences observed between groups are statistically significant because of the relatively small sample of people traveling from countries with more liberal abortion laws.

## Conclusions

Our study demonstrates that women and pregnant people from across Europe undertake burdensome travel to England and Wales for abortion care. Despite the differing legal and social contexts from which they traveled, the majority of participants in our study cited delays in care-seeking, detailed the logistical and financial implications of traveling, and indicated that the experience was difficult. Our findings suggest that it is crucial to improve access to information on abortion care and services in both restrictive and liberal settings across Europe. The results also highlight the importance of removing of all legal and procedural barriers that can delay access to abortion, particularly gestational limits. The removal of these barriers would ensure these governments can fulfill their human rights obligations and enable women and pregnant people to obtain necessary healthcare in their own communities without impediments. The failure to do so may result in reinforcing inequalities across Europe.

## Data Availability

Due to our commitment to protect the confidentiality and anonymity of those who received abortion services at BPAS, we cannot make the data used for this study publicly available for download. The data that support the findings of this study are available on request from the corresponding author.

## Abbreviations

ERC: European Research Council
BPAS: British Pregnancy Advisory Service
GA: Gestational Age

## Footnotes

1 In our recruitment materials for this study we used the word ‘women’ to describe those eligible to participate, we did not, however, survey participants on their gender identity. Because we cannot report on our participants gender identity and we acknowledge that not all people who are capable of pregnancy and/or desiring of abortion identify as women we will primarily use the gender-inclusive term “people” to refer to and acknowledge the experiences of women and any other pregnant people who participanted in our study. We will use gender-explicit terms (e.g. “women”) when reporting the results of other research studies, where participants identified as such.

2 Participants were asked to specify the currency that they reported costs in. Most participants reported costs in Euros, but two participants reported in British Pounds (conversion 1 GBP = .8928 EUR), one reported in Swedish Kroner (1 KR = 10.8 EUR), and one reported in Danish Krones (1 DKK = 7.46 EUR). Conversions were made based on rates in August of 2019.

3 Although we only recruited from clinics in England and Wales, we used “Great Britain”, or the “UK” in our survey instruments depending on the question so as to have standardized instruments across data collection sites and avoid confusing participants. We present our results with the terms used in each respective question.
