# Correction to: Cross-country abortion travel to England and Wales: results from a cross-sectional survey exploring people’s experiences crossing borders to obtain care

**DOI:** 10.1186/s12978-021-01271-z

**Published:** 2021-11-15

**Authors:** Camille Garnsey, Giulia Zanini, Silvia De Zordo, Joanna Mishtal, Alexandra Wollum, Caitlin Gerdts

**Affiliations:** 1 Ibis Reproductive Health, 1736 Franklin St, Suite 600, Oakland, CA 94612 USA; 2 Department of Anthropology, University of Barcelona, Montalegre, 6-8, 08001 Barcelona, Spain; 3 Department of Anthropology, University of Central Florida, 4297 Andromeda Loop, Orlando, FL 32816 USA

## Correction to: Reprod Health (2021) 18:103 https://doi.org/10.1186/s12978-021-01158-z

After publication of this article [1], the authors reported three adjustments:

- In Table 1, second column, the percentage of travelers from the Republic of Ireland should be “90%" instead of “98%”. The n (65) is correct.
- In Table 3, fourth column, the first “Prefer not to answer/no response” row should reflect the sum of the second and third columns, and needs to be "5(5%)" instead of "1(1%)".
- In Table 4, second column, there was an extraneous “0" in the “Cost of abortion procedure”-part after “I did not have to pay for my abortion”. This “0” should be removed.

The original article [1] has been updated.
